# Comprehensive analysis of the effect of etiolated tea cultivars and harvest seasons on volatile compounds and *in vitro* antioxidant capacity in steamed green teas

**DOI:** 10.1016/j.fochx.2024.101279

**Published:** 2024-03-16

**Authors:** Shuishan Mi, Shanjie Han, Mengxin Wang, Baoyu Han

**Affiliations:** a Zhejiang Provincial Key Laboratory of Biometrology and Inspection and Quarantine, College of Life Sciences, China Jiliang University, Hangzhou 310018, China; b Hangzhou Tea & Chrysanthemum Co. Ltd., Hangzhou, China

**Keywords:** Steamed green tea, Etiolated tea cultivars, Harvest season, Aroma characteristics, Odor activity value, Antioxidant capacity

## Abstract

To explore the effects of harvest seasons and etiolated cultivars on the volatile compounds of steamed green teas, this study analyzed comprehensively the volatile compounds of steamed green teas using simultaneous distillation extraction-gas chromatography–mass spectrometry (SDE-GC–MS) and chemometrics analytical techniques in combination with odor activity value (OAV). Additionally, the *in vitro* antioxidant capacity of the steamed green teas was evaluated. The results showed that 95 volatile compounds were identified, among which aldehydes, ketones, alcohols, and acids were the main types in steamed green teas made from etiolated tea cultivars. Furthermore, the relative content of volatile compounds in steamed green tea was significantly negatively correlated with season (*P* < 0.05). In steamed green teas harvested in different seasons, spring tea contained a higher abundance of volatile compounds such as (+)-δ-cadinene, farnesyl acetone, carvenone, trans-β-ionone, and nerolidol. The differences of total volatile compounds among the three etiolated tea cultivars were not remarkable (*P* > 0.05). Combined with the OAV, 20 and 19 key aroma compounds in steamed green teas from different harvest seasons and cultivars were identified, respectively, which could bring unique aromas to different steamed green tea samples. By comparison, summer tea had the strongest antioxidant capacity, while there was no obvious difference in the antioxidant capacity among cultivars. This study provided a scientific basis for the aroma quality of steamed green teas made from etiolated tea cultivars in different harvest seasons.

## Highlights

• 95 volatile compounds in steamed green tea samples made from three etiolated tea cultivars in different harvest seasons by SDE-GC–MS were identified.
• Variations of volatile compounds in steamed green teas from different harvest seasons and three etiolated tea cultivars were explored.
• Aroma characteristics of steamed green teas in different harvest seasons and three etiolated tea cultivars were confirmed.

## 1 Introduction

Known as the national drink in China, green tea belongs to the category of unfermented tea, with unique flavors and health benefits (Shi et al., 2022). It is the oldest, the most varieties, and the most widely tea, with output ranking first among the six basic tea types in China. According to the fixation and drying ways, green tea is subdivided into steamed green tea, pan-fried green tea, sun-dried green tea, and baked green tea (Shi et al., 2022). Among the various traditional processing techniques for Chinese green tea, the steamed green tea processing, which originated in the Tang and Song Dynasties, involves the use of high-temperature steam to denature the activity of enzymes in fresh tea leaves, thereby preserving the tea's freshness (e. g. Enshi Yulu tea and Yixing Yangxian tea). The quality characteristics of steamed green tea are different from those of other types of green teas. Studies have been conducted to compare the sensory quality and physicochemical properties of green tea subjected to four different processing methods (Shi et al., 2022; Wang et al., 2022) and comprehensively characterize the dynamics of non-volatile metabolites during the manufacturing of steamed green tea (Gui et al., 2023). Steamed green tea has a special aroma different from other types of green teas, but the systematic research on the volatile compound of steamed green tea remains unclear.

The aroma of tea is a crucial indicator of its quality, and a significant factor in determining its flavor and characteristics. Tea produced from different cultivars usually exhibit different flavors, known as “variety flavor” (Yue et al., 2023). Consumers' obsession with high-quality and unique flavors has prompted tea breeders to select high-flavor tea plants. Among these cultivars (Fig. S1), Huangjinya (*Camellia sinensis* cv. “Huangjinya”), a light-sensitive mutant tea cultivar with a yellow phenotype in fresh tea leaves, is widely cultivated in China because of its bright yellow colour and high amino acid content. Huanglongjin (*Camellia sinensis* cv. “Huanglongjin”) and Dahuangpao (*Camellia sinensis* cv. “Dahuangpao”) are newly cultivated etiolated tea cultivars, which belong to landraces, but are rare and special tea germplasms. They can not only be developed into characteristic and healthy tea products, but also have distinct landscape garden advantages, with promising prospects for planting and promotion. Therefore, the study of investigating the aromatic characteristics of different etiolated tea cultivars can provide a basis for the quality characteristics and utilization of specific tea cultivars.

In addition to the tea cultivar, the quality of tea aroma is also affected by various factors, such as harvest season, origin, and processing techniques. Among them, harvest season is a crucial factor that significantly affects the unique aroma of tea due to variations in the content of volatile compounds in tea (Zhang et al., 2022). Studies have suggested that six volatile compounds may be the key contributors to stronger flowery, fresh, and sweet aromas of spring-picked white tea than those of autumn-picked white tea (Zhang et al., 2022). Furthermore, 14 non-volatile characteristic compounds can be applied to identify the harvest season of white tea (Ma et al., 2022). It has been found that the autumn-picked Yingde black tea possessed a more intense aroma, and had strong correlations with floral, bitter, grass/green, sweet, and woody fragrance (Liu et al., 2021). Yin et al. (2022) found that the harvest season had an important influence on the flavor quality of Xinyang Maojian green tea. Nevertheless, the effects of harvest seasons on the aroma characteristics in steamed green tea and dynamic changes of volatile compounds have not been clarified yet, so it is necessary to perform a systematic study on the comparison of steamed green teas harvested in spring, summer, and autumn.

Up to now, multiple attempts have been made to extract and enrich the volatile compounds by application of different advanced techniques, such as simultaneous distillation extraction (SDE), solid-phase microextraction (SPME), solid-phase extraction (SPE), steam distillation under reduced pressure (DRP), solvent assisted flavor evaporation (SAFE), and so on. However, each technique imparts discrimination and incomplete recoveries for flavor compounds in tea. Although the SDE method is well known to produce artifacts, it is used to separate and concentrate tea volatile compounds in one step and to enhance the number and levels of volatile compounds, captured with the advantages of convenience, high extraction rate and good reproducibility, in addition, it avoids less volatile compounds that may chromatograph poorly in a GC (Yin, Kong, Liu, Wang, et al., 2022). Zhai et al. (2022) reported that the SDE technique was suitable for aroma analysis of tea. Sheibani et al. (2016) compared SDE with SPME for the separation of flavor compounds in Jin Xuan oolong tea and found that the number of identified volatile compounds in SDE was significantly higher. Additionally, the SDE procedure was similar to tea product brewing condition.

In this study, therefore, SDE-GC–MS was adopted to analyze the volatile compounds in steamed green teas of different cultivars in different harvest seasons, comprehensively explore the changes of volatile compounds in steamed green tea from different seasons and cultivars, reveal its aroma characteristics. Additionally, the total antioxidant capacity (T-AOC) and 1,1-diphenyl-2-picrylhydrazyl radical scavenging activity (DPPH) were carried out to evaluate their *in vitro* antioxidant capacity. The results of this study will provide valuable insights into the volatile compounds and main aroma in steamed green tea from different seasons and cultivars, thus providing a theoretical basis for the scientific evaluation of the aroma quality of etiolated tea cultivars.

## 2 Materials and methods

### 2.1 Materials and chemical reagents

The tea samples were all collected from the organic tea garden of Weizhong Family Farm in Songyang County, Lishui City, Zhejiang Province, and three etiolated tea cultivars were selected, namely Huangjinya, Huanglongjin, and Dahuangpao. All samples were picked with one bud and two leaves, harvested in spring, summer, and autumn, and processed with the same traditional steamed green tea processing technology. After production, all samples were collected in triplicate. The sample details are shown in Table S1. All samples were stored at −20 °C after collection.

Analytical grade anhydrous sodium sulfate was purchased from Sinopharm Chemical Reagent Co., Ltd. (Shanghai, China). Chromatographic grade absolute ether was purchased from Tedia Co., Inc. (Ohio, USA). Ethyl decanoate and n-alkanes (C4-C43) were purchased from Signa-Aldrich (Shanghai, China). Pure water was prepared using a Milli-Q water purification system (Millipore, Billerica, MA, USA).

### 2.2 Extraction of steamed green tea volatile compounds by SDE

The volatile compounds were extracted from the tea samples using SDE method with three replicates for each assay. The procedure of SDE followed the previous method used in our research with slight modification (Li et al., 2024). Each tea samples to be analyzed was weighed 50.00 g and placed in a round-bottom flask of 3000 mL capacity. Then, 1000 mL of boiling ultrapure water was added with 1 mL of 10^−4^ mg/mL ethyl caprate as an internal standard. The round-bottomed flask was connected to one side of the SDE device and heated to micro-boiling with an electric heating mantle. The other side of the SDE was connected to an extraction bottle containing 50 mL of anhydrous ether, and the extraction bottle was heated in a 50 °C water bath. Next, the condensate system was turned on. The timing began when the lower end of the condensation tube of the SDE device dripped continuously, and the electric heating mantle and water bath were turned off after 30 min. When there was no liquid drop from the condensation tube, the extraction bottle was removed, added with a small amount of anhydrous sodium sulfate and placed in a refrigerator at −20 °C for 24 h to remove a small amount of water. Finally, the extract was filtered and concentrated to 1 mL with high-purity nitrogen for GC–MS analysis.

### 2.3 GC–MS analysis

Samples were analyzed using Agilent GC–MS (Agilent 6890 A GC coupled with MSD 6975) (Agilent Technologies, Wilmington, DE, USA). The chromatographic column used in this study was the HP-5MS quartz capillary column (30.0 m × 250 μm × 0.25 μm). The GC inlet temperature was 250 °C. A carrier gas of 99.999% high-purity helium was used with a constant flow rate of 1.0 mL/min. The temperature program was as follows. The initial temperature was 50 °C for 5 min, then the temperature was increased to 190 °C at a rate of 3 °C/min and held for 5 min. The solvent was delayed for 3 min. The injector volume was 1 μL in splitless mode. The temperature of the electron impact (EI) ion source was 230 °C, with an ionization energy of 70 eV. The mass scan range was 30–100 *m*/*z*.

### 2.4 Qualitative and quantitative analysis of the steamed green tea volatile compounds

The volatile compounds in steamed green tea from different cultivars and harvest seasons were qualitatively analyzed by matching reference standards in the National Institute of Standards and Technology (NIST 11.L) to retention index (RI, determined by n-alkane C4-C43), and combining with the related literature on tea volatile compounds (Li et al., 2024; Shimoda et al., 1995). Using methods recommended by Shen et al. (2023) and He et al. (2023), the identified volatile compounds were semi-quantified using ethyl caprate as an internal standard. The formula was as follows:

$$C_{x}=\frac{A_{x}}{A_{i}}\times \mathrm{Ci}$$

C_x_: the relative content of the target volatile compound (μg/L); C_i_: the content of internal standard (μg/L); A_x_: the peak area of the target volatile compound; A_i_: the peak area of internal standard.

### 2.5 Odor activity values (OAVs) calculation

OAV is mainly utilized to characterize some key aroma-active compounds. The OAV of each volatile compound was calculated by dividing the content of each volatile compound by its odor threshold in water, and the threshold of the volatiles in water was referenced from other literature (Wang, Meng, et al., 2022; Yu et al., 2023). In general, volatile compounds with OAV > 1 are considered aroma-active compounds that contribute to the overall aroma of the samples.

### 2.6 Determination of tea polyphenols of steamed green tea

Adhering to national standards (GB/T 8313–2018), the quantification of tea polyphenols at 765 nm was conducted using the Folin-Ciocalteu method with gallic acid as the standard, employing the UV-1100 ultraviolet-visible (UV–vis) spectrophotometer (Mapada, Shanghai, China) (Fig. S2).

### 2.7 In vitro antioxidant capacity evaluation

T-AOC is an important indicator for evaluating the total antioxidant capacity of substances. The higher the T-AOC level, the stronger the antioxidant capacity (Zeng et al., 2018). T-AOC and DPPH assays were carried out to evaluate the *in vitro* antioxidant capacity of tea samples based on the methods described in Zeng et al. (2018). Tea sample (0.2 g) was weighed and extracted by 2 mL physiological saline or 2 mL 80% methanol, respectively. And then, the collected supernatants were collected after centrifugation for determination.

### 2.8 Statistical analysis

SPSS 23.0 statistical software (SPSS, Chicago, IL, USA) was utilized for the calculation of significant differences (*P* < 0.05) for volatile compounds, and SIMCA-P 14.1 software (Umetrics, Umea, Sweden) was utilized for principal component analysis (PCA) and orthogonal partial least squares-discriminant analysis (OPLS-DA). OriginPro 2022 (Origin Lab Corporation, Northampton, 313 Massachusetts, USA) was used for graphic drawing. The cluster analysis and heatmap analysis were performed using TBtools software. In addition, a correlation network diagram was prepared with the Cytoscape (version 3.9.1).

## 3 Results and discussion

### 3.1 Overall determination of volatile compounds in steamed green teas from three etiolated tea cultivars

**Fig. 1:**
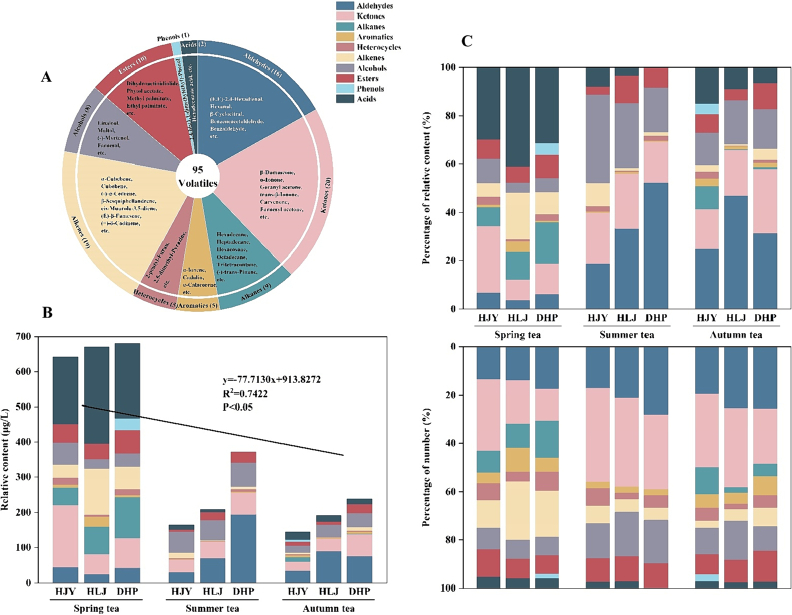
Types and relative content of volatile compounds in steamed green tea from different etiolated tea cultivars and seasons. (A) Categories of volatile compounds. (B) Total volatile compounds relative content and its correlation with different seasons. (C) The percentage of relative content and number of volatile compounds. (For interpretation of the references to colour in this figure legend, the reader is referred to the web version of this article.)

The tea aroma is a specific fragrance of tea formed by the combination of different aromatic substances in different concentrations on the olfactory nerve. In this study, the volatile compounds in steamed green tea samples made from three etiolated tea cultivars in different harvest seasons were detected and analyzed by means of SDE-GC–MS, and a total of 95 volatile compounds were identified. These volatile compounds were classified into 10 categories based on the nature of functional groups, including aldehydes, ketones, alkanes, aromatic hydrocarbons, heterocycles, alkenes, alcohols, esters, phenols, and acids (Fig. 1A, Table S2), among which ketones (20) were the largest number, contributing to 21.05% of the total volatile compounds, followed by alkenes (19), aldehydes (16), and esters (10), accounting for 20%, 16.84%, and 10.53% of the total volatile compounds numbers, respectively. Moreover, the number of these four types of compounds together accounted for 68.42% of that of the total volatile compounds. However, alkanes (9), alcohols (8), aromatic hydrocarbons (5) and heterocycles (5) were less in number, accounting for 9.47%, 8.42%, 5.26%, and 5.26%, respectively. Acids (2) and phenols (1) were the least in number, accounting for 2.11% and 1.05%.

As shown in Fig. 1B and C, the relative content and proportions of volatile compounds in the steamed green tea samples of three etiolated tea cultivars in different harvest seasons were investigated. The relative content of total volatile compounds in the steamed green tea samples of the three etiolated tea cultivars ranged from 143.94 to 680.06 μg/L, with the lowest level in autumn Huangjinya samples and the highest level in spring Dahuangpao samples. The comparison of the relative content of different volatile compounds revealed that aldehydes had the largest proportion of relative content (3.85%–52.40%), followed by ketones, alcohols, and acids, all with an average relative content of >15%, and the proportion of relative content of these four types of volatile compounds to the total volatile compounds was ranging from 55.41% to 92.82%. Accordingly, the proportions of average relative content of heterocycles, aromatic hydrocarbons, and phenolic compounds were the lowest (all below 2.0%). Notably, acids had a high relative content proportion but a small quantity (2), mainly because of the high relative content of hexadecanoic acid, up to 271.83 μg/L and accounting for 40.54% of the total volatile compounds content. Although there were many alkenes and esters, the proportion of their average relative content was below 10%, which was different from the composition of volatile compounds in other steamed green teas (Shi et al., 2022). It is probably because only the etiolated tea cultivars were used in this study, leading to the variability of the volatile compound composition. There were also some differences in the volatile compound composition characteristics between the steamed green tea in this study and other tea types. For example, Wuyi rock tea had the highest content of heterocycles in its aroma composition (Yue et al., 2023), black tea had the largest content of alcohols and aldehydes (Huang et al., 2022), while the aroma of compressed white tea was mainly composed of alcohols, aldehydes, and esters (Wang, Wang, et al., 2022), and the main volatile compounds of large-leaf yellow tea and small-leaf yellow tea in yellow tea were heterocycles and aromatics (Guo et al., 2019), alcohols and aldehydes (Dong et al., 2023), respectively. In this study, it was discovered that the volatile compounds in steamed green tea made from etiolated tea cultivars were primarily composed of aldehydes, ketones, alcohols, and acids, and in particular, aldehydes and ketones were higher in terms of volatile types and content, which was consistent with the volatile composition of steamed spring tea (SST) and steamed and baked spring tea (SBST) (Wang, Fu, et al., 2022).

### 3.2 Seasonal variation of volatile compounds in steamed green tea samples

**Fig. 2:**
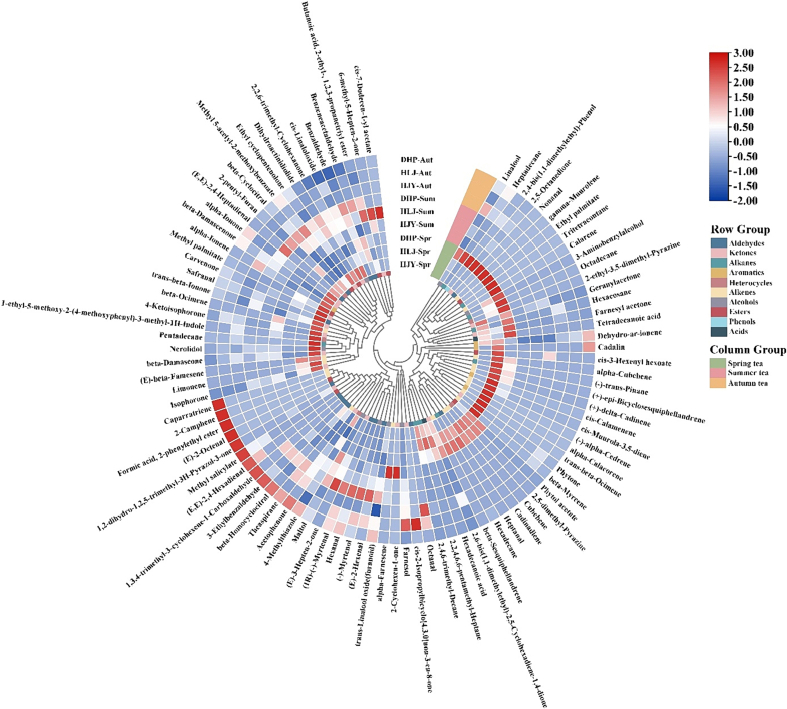
Heat map of volatile compounds in steamed green tea. (For interpretation of the references to colour in this figure legend, the reader is referred to the web version of this article.)

The changes in the relative content and composition of volatile compounds in steamed green tea in different seasons are displayed in Fig. 1B and 2. The relative content of in steamed green tea in spring, summer, and autumn ranged from 642.07 to 680.06 μg/L, 164.13 to 372.06 μg/L, and 143.94 to 239.17 μg/L, respectively. The relative content of volatile compounds in steamed green tea in spring was higher than that in summer and autumn, with significant differences (*P* < 0.05), while it was slightly higher in summer than in autumn, and the difference was not obvious (*P* > 0.05). Pearson's correlation analysis indicated that the relative content of volatile compounds in steamed green tea was significantly negative correlations with the season (month) (*P* < 0.05), and the results of linear regression suggested that the relative content of volatile compounds in steamed green tea decreased remarkably with the rise of month (Fig. 1B). According to previous studies (Liu et al., 2021; Zhang et al., 2022), tea aroma was greatly affected by seasons. The results of this study were consistent with the seasonal variation trend of volatile content in Xinyang Maojian (Yin, Kong, Liu, Jiang, et al., 2022) and high-latitude green tea (Wang, Li, et al., 2022).

**Fig. 3:**
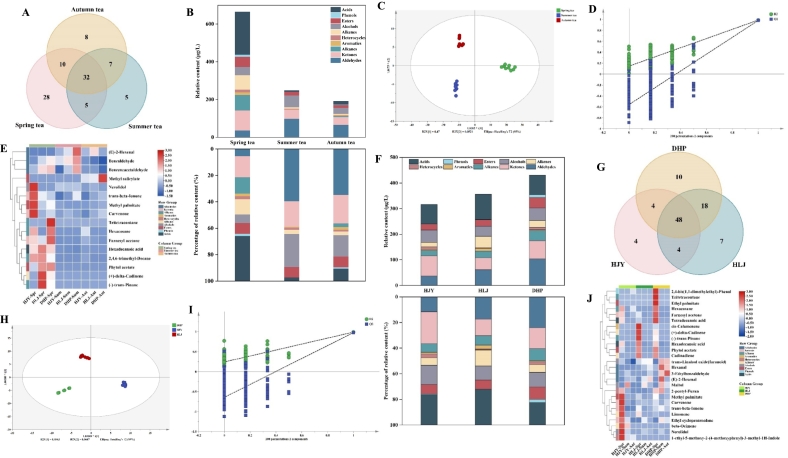
Characteristic of volatile compounds in steamed green tea from different seasons (A, B, C, D, E) and three etiolated tea cultivars (F, G, H, I, J). (A) Venn diagram. (B) Classification and percentage of volatile compounds. (C) OPLS-DA score plot. (D) Cross validation of OPLS-DA model. (E) Heatmap of 16 differential volatile compounds. (F) Classification and percentage of volatile compounds. (G) Venn diagram. (H) OPLS-DA score plot. (I) Cross validation of OPLS-DA model. (J) Heatmap of 26 differential volatile compounds. (For interpretation of the references to colour in this figure legend, the reader is referred to the web version of this article.)

The distribution of volatile compounds in steamed green tea in different harvest seasons was visualized by a Venn diagram (Fig. 3A). There were 75, 49 and 57 volatile compounds identified from spring tea, summer tea, and autumn tea, respectively. A total of 32 common volatile compounds in spring tea, summer tea, and autumn tea, and only 28, 5, and 8 volatile compounds detected in spring, summer and autumn tea, respectively. Evidently, there were a large number of unique volatile types in spring tea, with alkenes as the main unique substances. In terms of the classification of identified compounds (Fig. 3B), acids, ketones, alkanes and alkenes accounted for >70% of the total volatile compounds content in spring tea, with each category having a relative content of over 10%. In both summer and autumn tea, the volatile compounds with a relative content proportion above 10% were aldehydes, ketones and alcohols, which accounted for >70% of the total volatile compounds. The relative content proportion of alkenes and heterocyclics in spring tea was 11.53% and 2.21%, respectively, which decreased with the rise of months. On the contrary, ketones had the highest relative content proportion in autumn tea (21.49%). Compared with spring tea and autumn tea, summer tea exhibited the highest relative content proportions of aldehydes and alcohols, with no alkanes and phenols detected.

The highest relative content of acids in spring tea was attributed to the higher relative content of hexadecanoic acid (27.69%–40.54% of the total volatile compounds). Hexadecanoic acid is the major fatty acid in tea (Balaban et al., 2022) and the most abundant aroma component in dark tea (Lv et al., 2017), contributing significantly to the unique aroma of oolong tea (Wang et al., 2016), which is perceived sensorially as “slightly waxy/fatty” aroma. Compared with summer tea and autumn tea, spring tea had the highest relative content and types of alkenes, with higher average relative content of (+)-δ-cadinene (31.65 μg/L), cadinadiene (10.29 μg/L), and limonene (9.78 μg/L). (+)-δ-Cadinene has a “thyme/herbal/woody” aroma and presents high content in Indonesian black tea (Choi, 2017) and Xinyang Maojian (Yin, Wang, Kong, Zhu, et al., 2022), serving as the key odorant of Xinyang Maojian green tea (Yin, Kong, Liu, Jiang, et al., 2022). Cadinadiene has a fruity aroma, such as “spicy fruity mango” in the senses and high content in the essential oils of *Nepeta hindostana* (Siddique et al., 2019) and bee propolis (Balogun et al., 2021). Limonene is an important component of green tea and imparts a fresh lemon aroma to tea. Studies have uncovered that limonene content is closely related to the quality of tea (Baldermann et al., 2014). Summer tea and autumn tea possessed higher relative content proportions of aldehydes, ketones, and alcohols than spring tea. (E)-2-Hexenal and hexanal have the highest relative content of aldehydes in summer tea and autumn tea, respectively, and belong to green leaf volatiles (GLVs), with typical “green grassy” aroma, making a significant contribution to tea aroma (Chen et al., 2022) and effectively attracting pests in tea gardens. Trans-β-Ionone has a “floral/sweet/berry-like/violet-like” aroma, which also makes a great contribution to the tea aroma (Wang et al., 2020), with higher relative content in spring tea, summer tea, and autumn tea, showing a decreasing trend. Farnesyl acetone and carvenone have “floral/fruity/spearmint” aromas, with relatively high content in spring tea, significantly higher than that in summer and autumn tea (*P* < 0.05). The relatively high content of nerolidol and linalool in spring tea contributes to the pleasant aroma of “floral/green/sweet”, which was considered one of the key indicators of the quality of oolong tea, and the content of nerolidol was positively correlated with the grade of oolong tea (Zeng et al., 2023). Maltol was abundant in summer and autumn tea but absent in spring tea, and it was also identified in Japanese green tea and Bigelow green tea (Alluhayb & Logue, 2017; Kumazawa & Masuda, 1999) and regarded as a potent odorant.

In order to better compare the differences in the aroma of steamed green tea in different harvest seasons, an OPLS-DA model was developed based on the relative content of volatile compounds in steamed green tea. The results showed that this method was effective in distinguishing between steamed green teas samples in different harvest seasons (Fig. 3C), where the R^2^X, R^2^Y, and Q^2^ were 0.810, 0.977, and 0.962, respectively. The cross-validation demonstrated that the model was not over-fitted (Fig. 3D). Based on the criteria of variable importance in the projection (VIP) > 1 and *P* < 0.05, sixteen key volatile compounds were screened. On this basis, the heat map was used to visualize the differences in these volatile compounds (Fig. 3E). In contrast, spring tea had higher levels of (+)-δ-cadinene, farnesyl acetone, carvenone, trans-β-ionone, nerolidol, and (−)-trans-pinane, contributing to their “floral/green/herbal/fruity/fresh/sweet” aromas. Summer and autumn tea had higher content of volatile compounds with “green/grassy/minty” aromas, such as (E)-2-hexenal, benzaldehyde, and methyl salicylate *etc.*

### 3.3 Changes of volatile compounds in steamed green tea samples from different etiolated tea cultivars

Fig. 3F illustrated the changes in the content and composition of volatile compounds in steamed green tea made from different etiolated tea cultivars. The average relative content of in steamed green tea made from Huangjinya, Huanglongjin, and Dahuangpao was 316.71 μg/L, 356.71 μg/L, and 430.43 μg/L, respectively. The relative content of volatile compounds in the steamed green tea made from Dahuangpao was higher than that from Huanglongjin and Huangjinya, and it was higher from Huanglongjin than that from Huangjinya, with no significant differences (*P* > 0.05). A total of 60, 77, and 80 volatile compounds were identified in the steamed green tea made from Huangjinya, Huanglongjin, and Dahuangpao, respectively. As shown in Fig. 3G, there were 48 common volatile compounds in these three etiolated cultivars, and only 4, 7, and 10 volatile compounds were detected from Huangjinya, Huanglongjin, and Dahuangpao, respectively, suggesting that these three etiolated cultivars contain similar volatile compounds. Based on the classification of identified volatile compounds (Fig. 3F), the relative content proportions of aldehydes, ketones, alcohols, and acids in volatile compounds were all above 10% from Huangjinya and Dahuangpao, which constituted >69% of the total volatile compounds, whereas these four classes of compounds, along with alkenes, constituted over 80% of the total volatile compounds from Huanglongjin. The high content of alkenes in Huanglongjin was attributed to the remarkably high relative content of (+)-δ-cadinene, up to 65.17 μg/L (Table S2). Besides, acid content proportion in the volatile compounds of Huanglongjin was the highest, although the difference from Huangjinya was not significant. In contrast, aldehydes had the highest content proportion in Dahuangpao, while ketones and alcohols had the highest content proportions in Huangjinya.

Compared with Huangjinya and Huanglongjin, Dahuangpao possessed the largest number and highest content of aldehydes. (E)-2-Hexenal had the highest relative content of aldehyde volatiles in the three etiolated tea cultivars, accounting for >30%. Benzaldehyde, benzeneacetaldehyde, and β-cyclocitral accounted for over 5% of the aldehyde volatiles in all three cultivars, with aroma notes of “almond-like/floral/fresh/rose-like”. Six alkenes were only identified from Huangjinya, among which limonene had the highest relative content. There were 14 terpenes identified from both Huanglongjin and Dahuangpao, among which (+)-δ-cadinene and cadinadiene had the content proportions of >35% and 10%, respectively. Notably, theaspirane imparted a “tea/green/herbal/fresh” aroma, and its content proportion exceeded 10% in both Huangjinya and Huanglongjin. Theaspirane was found to be a key active aromatic compound unique to steamed green tea in the study on differences of volatiles in pan-fried green tea, baked green tea, steamed green tea, and sun-dried green tea (Shi et al., 2022). β-Ocimene (“floral”) was identified only in Huangjinya, accounting for about one-third of the alkenes in Huangjinya. The content proportion of trans-β-ionone in the ketones of all three etiolated cultivars was above 20%. Meanwhile, the content proportions of farnesyl acetone, carvenone, and ethyl cyclopentenolone were higher than 10%, which provided floral and fruity aroma for the steamed green tea made from the three etiolated cultivars. Linalool, maltol, trans-linalool oxide (furanoid), and nerolidol generally had proportions of over 10% among the alcohols of three etiolated cultivars, which were able to provide a floral aroma. Liu et al. (2023) found that linalool, trans-linalool oxide (furanoid), and trans-β-ionone were key contributing volatile compounds for the floral aroma of green tea.

For the purpose of revealing the difference of volatile compounds in steamed green teas made from etiolated tea cultivars and measuring the contribution of each variable to classification, the OPLS-DA model was established, in which volatile compounds with VIP >1 were considered to have significant contributions to classification. As shown in the Fig. 3H, steamed green teas samples made from different etiolated tea cultivars showed a clear separation, where the R^2^X, R^2^Y, and Q^2^ were 0.981, 0.987, and 0.969, respectively. The permutation test manifested that the model was not over-fitted (Fig. 3I). Based on the VIP > 1, 26 key volatile compounds were screened. On this basis, a heat map was plotted to visualize the differences in these volatile compounds (Fig. 3J). By comparison, Huangjinya had higher content of volatile compounds with a floral aroma, including linalool, trans-β-ionone, carvenone, nerolidol, and β-ocimene. Huanglongjin had high content of volatile compounds with herbaceous and woody aromas, e, g. (+)-δ-cadinene, (−)-trans-pinane, and cis-calamenene. Dahuangpao had higher content of volatile compounds with floral, fruity, and fresh aromas, such as farnesyl acetone, trans-linalool oxide (furanoid), ethyl palmitate, (E)-2-hexenal, and hexanal.

### 3.4 Key aroma characteristic of steamed green tea samples from different harvest seasons and etiolated tea cultivars

**Fig. 4:**
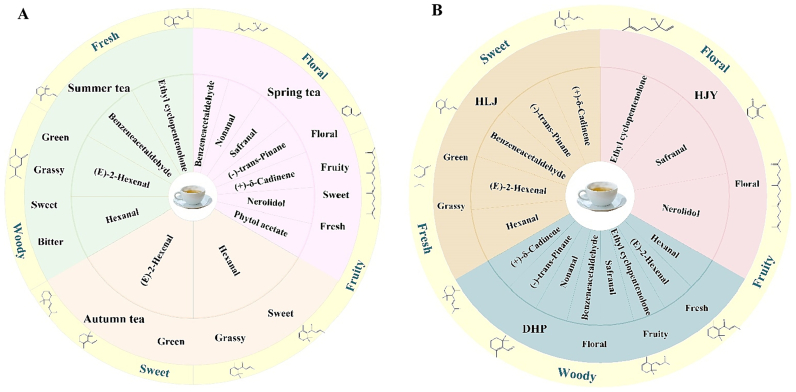
The enriched and contributed key volatiles compounds in steamed green tea. (A) 20 key volatiles compounds from different seasons. (B) 19 key volatiles compounds from different etiolated tea cultivars. (For interpretation of the references to colour in this figure legend, the reader is referred to the web version of this article.)

The contribution of volatile compounds to the overall aroma of steamed green tea was evaluated through OAV. The volatile compounds with OAV > 1 are considered key aroma-active compounds, while those with OAV > 10 are believed to make a significant contribution to the overall aroma of the tea (He et al., 2023; Shi et al., 2022). Volatile compounds with higher OAVs are more critical to the formation of tea aroma quality (Shi et al., 2022; Yin, Wang, Kong, Zhu, et al., 2022). In this study, 15 volatile compounds with OAVs >1 in steamed green tea from different harvest seasons were screened (Table 1). There were seven volatile compounds with OAVs >1 in spring tea, summer tea, and autumn tea samples, namely, β-cyclocitral, β-homocyclocitral, β-damascenone, β-damascone, α-ionone, trans-β-ionone, and linalool, among which β-damascenone, β-damascone, and trans-β-ionone had an OAV markedly higher than 100. These three volatile compounds have relatively low odor thresholds, which can enhance the floral and fruity aroma of steamed green tea. In particular, trans-β-ionone has higher relative content and plays an essential role in the formation of tea aroma. In contrast, there were only four volatile compounds with OAVs >1 in the spring tea samples, including nonanal (floral/citrus), safranal (floral/sweet/herb), (+)-δ-cadinene (herbal/woody), and nerolidol (floral/green). The OAV of ethyl cyclopentenolone (sweet/caramellic) was >1 in the summer tea samples only. Hexanal and (E)-2-hexenal had OAVs >1 in summer and autumn tea, indicating a strong intensity of grassy aroma in summer and autumn tea. Benzeneacetaldehyde (rose-like/floral/sweet) had OAVs>1 only in spring and summer tea. However, some volatile compounds still have unknown thresholds, and their intensity and concentration change disproportionately. Therefore, in addition to the volatile compounds of OAVs >1, the differential volatile compounds and odor descriptions with VIP >1 were selected. Ultimately, 20 representative volatile compounds were screened. According to the Fig. 4A, spring tea had richer aromas, dominated by floral and fruity aroma, with some fresh notes. The aromas of summer tea and autumn tea tended to be grassy with some sweetness, especially a slight bitterness in the sweetness of summer tea. Some aroma profiles have demonstrated that autumn tea of black tea had richer aroma attributes (Liu et al., 2021), which is inconsistent with the results of this study, probably due to the influences of processing technologies, tea cultivars, extraction methods, and detection methods.

**Table 1:** Key aroma compounds of steamed green tea from different harvested seasons and etiolated tea cultivars (OAV > 1).

No.	Volatile compounds	Odor description^B^	Average relative content (μg/L)						OT^A^(μg/L)	OAV					
			Spring tea	Summer tea	Autumn tea	HJY	HLJ	DHP		Spring tea	Summer tea	Autumn tea	HJY	HLJ	DHP
1	Hexanal	Fresh, Green, Grass, Fruity	2.26	14.16	12.41	–	7.07	21.76	4.5	0.50	3.15	2.76	–	1.57	4.83
2	(E)-2-Hexenal	Green, Fresh	0.28	47.12	28.98	11.91	29.65	34.82	17	0.02	2.77	1.70	0.70	1.74	2.05
3	Benzeneacetaldehyde	Green, Sweet, Floral	6.90	4.80	1.44	1.94	4.86	6.34	4	1.73	1.20	0.36	0.49	1.21	1.59
4	Nonanal	Fresh, Fruity	2.89	–	–	–	–	2.89	1	2.89	–	–	–	–	2.89
5	Safranal	Fresh, Sweet, Floral	5.23	2.46	2.81	4.78	2.64	3.07	3	1.74	0.82	0.94	1.59	0.88	1.02
6	β-Cyclocitral	Fresh, Sweet, Fruity	5.87	8.72	6.22	7.54	5.75	7.52	5	1.17	1.74	1.24	1.51	1.15	1.50
7	β-Homocyclocitral	Fresh, Woody	2.29	2.09	2.56	1.79	1.75	3.40	0.2	11.47	10.43	12.78	8.93	8.74	17.01
8	Ethyl cyclopentenolone	Sweet, Bitter	9.95	14.42	5.82	13.74	6.32	10.13	10	1.00	1.44	0.58	1.37	0.63	1.01
9	β-Damascenone	Woody, Sweet, Fruity, Floral	1.54	1.10	1.78	2.36	0.59	1.47	0.002	768.85	551.32	890.71	1177.75	297.09	736.03
10	β-Damascone	Fruity, Floral, Sweet	2.40	1.79	1.03	3.87	1.03	0.31	0.002	1200.06	893.35	512.96	1937.02	516.56	152.79
11	α-Ionone	Sweet, Woody, Floral, Fruity	4.09	2.57	2.54	3.77	1.65	3.78	0.4	10.23	6.42	6.35	9.43	4.12	9.46
12	trans-β-Ionone	Sweet, Fruity, Woody, Floral	26.98	10.85	9.81	17.12	13.43	17.09	0.007	3853.93	1549.62	1401.61	2445.87	1918.05	2441.23
13	(+)-δ-Cadinene	Thyme, Fresh, Woody	31.65	–	–	0.63	21.72	9.31	1.5	21.10	–	–	0.42	14.48	6.20
14	Linalool	Fruity, Floral	11.92	6.10	10.69	9.53	7.44	11.75	6	1.99	1.02	1.78	1.59	1.24	1.96
15	Nerolidol	Floral, sweet	14.44	6.60	0.96	16.85	2.99	2.16	10	1.44	0.66	0.10	1.69	0.30	0.22

Note: OT: Odor thresholds in water. The values were calculated according to reported references.

A: (Wang et al., 2022; Yu et al., 2023). B: https://www.thegoodscentscompany.com; https://www.flavornet.org/flavornet.html.

As shown in Table 1, 15 volatile compounds with OAVs >1 were screened in steamed green tea samples of three etiolated cultivars, namely Huangjinya, Huanglongjin, and Dahuangpao. There were 7 volatile compounds with OAVs >1 in these three etiolated cultivars, identical to the volatile compounds with OAVs >1 in different harvest seasons, which provided the basic aroma of “floral/fruity/sweet/woody/fresh”. The OAV of nerolidol was >1 only in Huangjinya, while that of nonanal exceeded 1 only in Dahuangpao. Hexanal, (E)-2-hexenal, benzeneacetaldehyde, and (+)-δ-cadinene were the volatiles with OAVs >1 in Huanglongjin and Dahuangbao only. The OAVs of safranal and ethyl cyclopentenolone were >1 in Huangjinya and Dahuangbao only. On this basis, the volatile compounds with high content, or low threshold and odor description were also selected from the differential volatile compounds with VIP >1. Finally, 19 representative aroma compounds were finally identified (Fig. 4B). Besides the basic aroma, Huangjinya mainly had a floral aroma, Huanglongjin mainly had herbaceous and woody aromas, and Dahuangpao had stronger floral and fruity aromas and a fresh aroma. Yang et al. (2022) researched that 11 key aroma compounds were screened from Huangjinya by OAV identification, and the aroma of these compounds was mainly floral, which was consistent with the results of this study.

### 3.5 Antioxidant capacity of steamed green tea samples from different harvest seasons and etiolated tea cultivars

Tea is widely known as a good dietary source of natural antioxidants, mainly due to polyphenolic compounds. However, the volatile compounds in green tea, which have been more frequently studied in the past, should also be considered. Tea aroma is one of the important factors affecting the quality of tea. Aroma has been used in folk therapies since ancient times, and now aromatherapy is becoming increasingly popular, with calming, tranquilizing and other physiological effects. Studies have denoted that the aroma of green tea could significantly improve the serum antioxidant capacity of mice, which proves its antioxidant activity (Li et al., 2017).

**Fig. 5:**
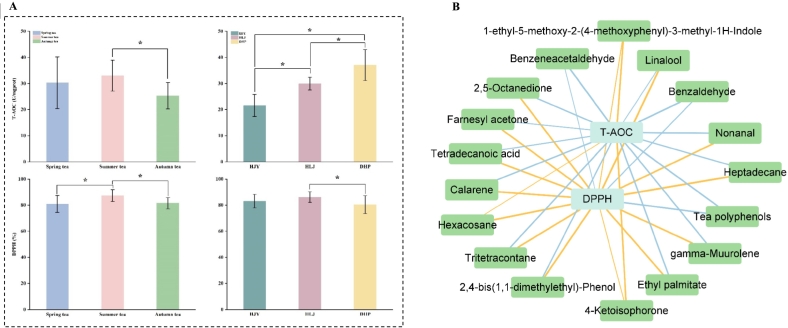
Antioxidant capacity analysis of steamed green tea. (A) The *in vitro* antioxidant capacity of steamed green tea from different seasons and etiolated tea cultivars determined by T-AOC and DPPH. * indicate signification differences (*P* < 0.05). (B) Correlation network of volatile compounds, tea polyphenols, and the *in vitro* antioxidant capacity of T-AOC and DPPH in steamed green tea. Blue squares represent antioxidant indexes; green squares volatile compounds. Line thickness represents the strength of correlation, and line colour represents the positive (blue) or negative (orange) correlation. (For interpretation of the references to colour in this figure legend, the reader is referred to the web version of this article.)

As shown in Fig. 5A, T-AOC and DPPH were used to evaluate the *in vitro* antioxidant capacity of steamed green tea made from different etiolated cultivars in different harvest seasons. One-way analysis of variance showed that summer tea had the strongest total antioxidant capacity, higher than spring tea (*P* > 0.05) and significantly higher than autumn tea (*P* < 0.05), but there was no significant difference between spring tea and autumn tea (*P* > 0.05). Dahuangpao had the strongest total antioxidant capacity, which was prominently higher than that of the other two cultivars (*P* < 0.05), followed by Huanglongjin, which was significantly higher than that of Huangjinya (*P* < 0.05). It has been discovered that among green tea in different seasons, summer tea showed the strongest total antioxidant capacity, followed by spring tea and autumn tea (Pan et al., 2017), which was in line with the findings of this study. In the aspect of DPPH, the DPPH of summer tea was evidently higher than that of autumn tea and spring tea (*P* < 0.05), but it was not overtly different between autumn tea and spring tea (*P* > 0.05). Moreover, Huanglongjin had higher DPPH than Huangjinya (*P* > 0.05) and significantly higher DPPH than Dahuangpao (*P* < 0.05), but there was no significant difference between Huangjinya and Dahuangpao (*P* > 0.05). Ma et al. (2022) revealed that for white tea in different harvest seasons, the DPPH of summer tea was the lowest, which was clearly different from that of spring tea and autumn tea (*P* < 0.05), opposite to the results in this study. The possible reason involves the impacts of cultivars, processing technologies, *etc.*

Tea polyphenols are the most important antioxidant compounds in tea, in addition, studies confirmed that the aroma of green tea had antioxidant activity (Li et al., 2017). Therefore, to further analyze the relationship between the volatile compounds, tea polyphenols, and antioxidant capacity in steamed green tea, Pearson's correlation analysis (Fig. 5B) was conducted for volatile compounds, tea polyphenols and antioxidant capacity. Tea polyphenols were significantly positively correlated with T-AOC and DPPH (*P* < 0.05). For volatile compounds, there were four volatile compounds significantly correlated with the T-AOC, among which benzaldehyde and benzeneacetaldehyde had significantly positive correlations (*P* < 0.05). 12 volatile compounds were significantly negatively associated with DPPH (*P* < 0.05). However, the relationship between these volatile compounds and antioxidant capacity was only preliminarily investigated through correlation in this study. In the future, systematic and in-depth work is needed to explore the biological effects of these aromatic compounds in order to fully understand the physiological effects of tea aroma.

## 4 Conclusion

A total of 95 volatile compounds in steamed green tea from different etiolated cultivars and harvest seasons were identified through SDE-GC–MS analysis. Aldehydes, ketones, alcohols, and acids were the main types of volatile compounds in steamed green tea made from etiolated tea cultivars, among which aldehydes and ketones were the most abundant in terms of both number and content. Pearson's correlation analysis showed that the relative content of volatile compounds in steamed green tea exhibited significantly negative correlations with the season (month). Spring tea contained a higher abundance of compounds with “floral/green/herbal/fruity/fresh/sweet” aromas, while summer tea and autumn tea had higher levels of volatile compounds with “green/grassy/minty” aromas. According to OPLS-DA analysis, Huangjinya had higher content of compounds with a floral aroma, Huanglongjin had higher content of compounds with herbaceous and woody aromas, and Dahuangpao had higher content of volatile compounds with floral, fruity, and fresh aromas. 20 and 19 key aroma compounds in steamed green teas from different harvest seasons and etiolated cultivars were screened, respectively, which could provide a unique aroma to various steamed green tea samples. Correlation analysis indicated that 4 and 12 volatile compounds were significantly related to T-AOC and DPPH, respectively. Although this study provided comprehensive information on the changes of volatile compounds in steamed green teas from different harvest seasons and etiolated cultivars, further systematic studies concerning precise quantitative and molecular sensory science are needed to explore the influence of key compounds on the flavor and health benefits of tea made from etiolated cultivar.

## Ethics approval

The study did not involve any human or animal testing.

## CRediT authorship contribution statement

**Shuishan Mi:** Data curation, Formal analysis, Methodology, Software, Writing – original draft. **Shanjie Han:** Data curation, Formal analysis, Software, Validation, Writing – original draft. **Mengxin Wang:** Conceptualization, Funding acquisition, Methodology, Writing – review & editing. **Baoyu Han:** Conceptualization, Funding acquisition, Methodology, Supervision, Writing – review & editing.

## Declaration of competing interest

The authors declare that they have no known competing financial interests or personal relationships that could have appeared to influence the work reported in this paper.

## Data Availability

Data will be made available on request.

## Appendix A Supplementary data

Supplementary material Supplementary Table S1: The detailed information of experimental samples. Table S2: The relative content (μg/L) of volatile compounds in steamed green tea samples. Fig. S1: The external morphology of Huangjinya, Huanglongjin, and Dahuangpao. Fig. S2: The content of tea polyphenols in steamed green tea from different etiolated tea cultivars and seasons. Image 1
