# Supplementary material for: Comprehensive analysis of the effect of etiolated tea cultivars and harvest seasons on volatile compounds and in vitro antioxidant capacity in steamed green teas
Source: Food Chem X. 2024 Mar 16;22:101279. doi: 10.1016/j.fochx.2024.101279 (PMC10973806; doi:10.1016/j.fochx.2024.101279)
Supplement: Supplementary file 1 — Supplementary material Supplementary Table S1: The detailed information of experimental samples. Table S2: The relative content (μg/L) of volatile compounds in steamed green tea samples. Fig. S1: The external morphology of Huangjinya, Huanglongjin, and Dahuangpao. Fig. S2: The content of tea polyphenols in steamed green tea from different etiolated tea cultivars and seasons. [file mmc1.docx]

**Table S1** The detailed information of experimental samples.

| Sample No. | Harvested season | Tea cultivar | Standard for picking fresh leaves | Number of samples | Production area |
| --- | --- | --- | --- | --- | --- |
| 1 | Spring  2022.04.15 | Huangjinya (HJY) | One bud and two leaves | 3 | Songyang county, Lishui city, Zhejiang province, China |
| 2 |  | Honglongjin (HLJ) |  | 3 |  |
| 3 |  | Dahuangpao (DHP) |  | 3 |  |
| 4 | Summer  2022.07.15 | Huangjinya (HJY) |  | 3 |  |
| 5 |  | Honglongjin (HLJ) |  | 3 |  |
| 6 |  | Dahuangpao (DHP) |  | 3 |  |
| 7 | Autumn  2022.10.15 | Huangjinya (HJY) |  | 3 |  |
| 8 |  | Honglongjin (HLJ) |  | 3 |  |
| 9 |  | Dahuangpao (DHP) |  | 3 |  |

**Table S2** The relative content (μg/L) of volatile compounds in steamed green tea samples.

| **No.** | | **Volatile compounds** | **CAS** | **Odor description^A^** | **Spring tea** | | | **Summer tea** | | | **Autumn tea** | | |
| --- | --- | --- | --- | --- | --- | --- | --- | --- | --- | --- | --- | --- | --- |
|  |  |  |  |  | **HJY** | **HLJ** | **DHP** | **HJY** | **HLJ** | **DHP** | **HJY** | **HLJ** | **DHP** |
| **Aldehydes** | | | | | | | | | | | | | |
| 1 | Hexanal | | 66-25-1 | Green, Grassy, Fruity, Fresh | - | 1.55±0.15e | 5.24±0.53d | - | 6.47±0.63d | 36.00±3.55a | - | 13.21±1.29c | 24.03±2.75b |
| 2 | (E)-2-Hexenal | | 6728-26-3 | Green, Fresh, Fruity | - | - | 0.84±0.84e | 16.97±1.62d | 33.85±3.52c | 90.54±8.92a | 18.76±2.22d | 55.11±5.38b | 13.08±1.47d |
| 3 | Heptanal | | 111-71-7 | Citrus, Green, Fresh | - | 2.62±0.25b | 3.44±0.33a | - | - | - | - | - | - |
| 4 | (E,E)-2,4-Hexadienal | | 142-83-6 | Green, Fruity, Sweet, Floral | - | - | - | - | - | - | - | 2.11±0.26b | 3.40±0.38a |
| 5 | Benzaldehyde | | 100-52-7 | Cherry, Sweet, Bitter | 3.01±0.29d | 6.50±0.62c | 9.08±0.87b | 6.86±0.66c | 9.55±0.97b | 12.46±1.23a | 3.02±0.36d | 1.68±0.16d | - |
| 6 | Octanal | | 124-13-0 | Fatty, Fruity, Green, Fresh | - | - | - | - | - | - | 1.36±0.16a | 0.78±0.76b | - |
| 7 | (E,E)-2,4-Heptadienal | | 4313-03-5 | Fatty, Green, Oily | 9.58±0.92b | - | - | - | - | 12.05±1.13a | - | - | - |
| 8 | Benzeneacetaldehyde | | 122-78-1 | Floral, Sweet, Green | 3.69±0.35c | 8.06±0.77b | 8.96±0.85ab | - | 4.34±0.44c | 10.07±0.95a | 2.14±0.25d | 2.18±0.21d | - |
| 9 | (E)-2-Octenal | | 2548-87-0 | Green, Pungent, Spicy, Fresh | - | - | - | - | - | - | - | - | 3.81±0.43a |
| 10 | Nonanal | | 124-19-6 | Fruity, Floral, Fresh, Green | - | - | 8.66±0.83a | - | - | - | - | - | - |
| 11 | 3-Ethylbenzaldehyde | | 34246-54-3 | Almond-Like | - | - | - | 1.87±0.18c | - | 6.04±0.57b | - | 2.06±0.24c | 9.96±1.12a |
| 12 | Safranal | | 116-26-7 | Herbal, Sweety, Floral, Fresh | 11.01±1.55a | 2.57±0.25bc | 2.09±0.20c | 1.63±0.16c | 2.03±0.21c | 3.70±0.35b | 1.70±0.22c | 3.31±0.38b | 3.43±0.39b |
| 13 | β-Cyclocitral | | 432-25-7 | Fresh, Sweet, Green, Fruity | 12.87±1.23a | 2.34±0.22e | 2.40±0.23e | 1.83±0.17e | 10.56±1.11b | 13.77±1.30a | 7.91±0.94c | 4.35±0.52d | 6.39±0.72c |
| 14 | β-Homocyclocitral | | 472-66-2 | Woody, Fruity | 3.48±0.33b | 2.16±0.26c | 1.25±0.12de | 0.92±0.86e | 1.62±0.18cd | 3.72±0.33b | 0.96±0.11de | 1.47±0.17cde | 5.24±0.59a |
| 15 | (1R)-(-)-Myrtenal | | 18486-69-6 | Sweet, Cinnamon, Spicy | - | - | - | 0.71±0.66d | 1.32±0.11c | 5.27±0.45a | - | 3.47±0.40b | 3.27±0.33b |
| 16 | 1,3,4-trimethyl-3-cyclohexene-1-Carboxaldehyde | | 40702-26-9 |  | - | - | - | - | - | 1.34±0.12b | - | - | 2.43±0.24a |
| **Ketones** | | | | | | | | | | | | | |
| 17 | (E)-3-Hepten-2-one | | 5609-09-6 | Fruity, Spicy, Grassy | - | - | - | 0.51±0.47c | 0.64±0.54b | 1.72±0.15a | - | - | - |
| 18 | 2,5-Octanedione | | 3214-41-3 |  | - | - | 8.18±0.79a | - | 0.74±0.63b | - | 0.82±0.97b | 0.37±0.43bc | - |
| 19 | 6-methyl-5-Hepten-2-one | | 110-93-0 | Nutty, Roasty, Fruity | - | - | - | 1.00±0.93b | 2.07±0.18a | - | - | - | - |
| 20 | 2-Cyclohexen-1-one | | 930-68-7 | Roasted, Savory, Green | - | - | - | 2.23±0.29a | - | - | - | - | - |
| 21 | 2,2,6-trimethyl-Cyclohexanone | | 2408-37-9 | Pungent, Honey | 4.07±0.35a | 1.09±0.14d | - | 1.67±0.16c | 2.19±0.19b | 2.28±0.20b | 0.93±0.11d | 0.68±0.78d | - |
| 22 | Acetophenone | | 98-86-2 | Floral, Sweet | - | - | - | 1.37±0.13d | 2.60±0.23c | 2.66±0.23c | 3.41±0.43b | 0.79±0.92d | 5.22±0.53a |
| 23 | Isophorone | | 78-59-1 | Woody, Green, Fruity, Sweet | 2.42±0.28a | 1.22±0.12b | - | 0.93±0.87c | 0.57±0.46d | 0.76±0.69cd | - | - | - |
| 24 | 4-Ketoisophorone | | 1125-21-9 | Woody, Fruity, Sweet | 1.55±0.13a | - | - | 0.53±0.50b | - | - | 0.29±0.35c | 0.38±0.44c | - |
| 25 | Ethyl cyclopentenolone | | 21835-01-8 | Sweet, Caramellic | 29.86±2.57a | - | - | 11.37±1.50bc | 13.72±1.98bc | 18.17±1.46b | - | 5.24±0.64cd | 12.23±1.25bc |
| 26 | β-Damascenone | | 23696-85-7 | Floral, Fruit, Woody, Sweet | 4.61±0.40a | - | - | 0.53±0.45d | 0.82±0.65d | 1.96±0.16c | 1.92±0.23c | 0.97±0.11d | 2.46±0.26b |
| 27 | β-Damascone | | 35044-68-9 | Fruity, Floral, Sweet | 7.20±0.62a | - | - | 2.28±0.19b | 2.17±0.17b | 0.92±0.74c | 2.15±0.25b | 0.93±0.17c | - |
| 28 | α-Ionone | | 127-41-3 | Sweet, Woody, Floral | 9.47±0.81a | 1.70±0.16de | 1.12±0.17e | 0.96±0.82e | 2.34±0.19d | 4.41±0.35c | 0.89±0.15e | 0.90±0.14e | 5.83±0.60b |
| 29 | 1,2-dihydro-1,2,5-trimethyl-3H-Pyrazol-3-one | |  |  | 2.93±0.25b | - | - | 1.60±0.14c | - | - | - | - | 11.88±1.14a |
| 30 | Geranyl acetone | | 689-67-8 | Green, Fresh, Floral, Fruity | 11.79±1.14a | 3.60±0.34cde | 10.18±0.98b | 2.94±0.25def | 4.17±0.36cd | 4.95±0.40c | 1.96±0.23f | 2.40±0.28ef | 2.15±0.27f |
| 31 | trans-β-Ionone | | 79-77-6 | Floral, Sweet, Fruity, Woody | 43.58±3.75a | 21.17±2.20b | 16.18±1.54c | 3.25±0.28g | 8.36±0.71ef | 20.93±1.69b | 4.53±0.54fg | 10.75±1.24de | 14.15±1.36cd |
| 32 | cis-2-Isopropylbicyclo[4.3.0]non-3-en-8-one | |  |  | - | - | - | - | - | - | - | 4.48±0.52a | - |
| 33 | 2,6-Di-tert-butyl-p-benzoquinone | | 719-22-2 |  | 3.08±0.27b | 2.91±0.28b | 4.01±0.38a | - | - | - | 2.11±0.25c | - | - |
| 34 | Carvenone | | 499-74-1 | Spearmint | 35.90±3.89a | 5.52±0.53cd | 10.25±0.98b | 3.05±0.26d | 5.87±0.52c | 3.33±0.27cd | - | 4.87±0.56cd | 8.76±0.84b |
| 35 | Phytone | | 16825-16-4 | Fat | - | 6.01±0.57a | - | - | - | - | - | 1.12±0.13b | - |
| 36 | Farnesyl acetone | | 762-29-8 | Fruity, Floral | 21.01±1.87b | 11.76±1.12c | 34.88±3.33a | 0.51±0.43e | 0.89±0.76e | 0.63±0.57e | 4.76±0.56d | 2.10±1.79de | 0.86±0.83e |
| **Alkanes** | | | | | | | | | | | | | |
| 37 | 2,2,4,6,6-pentamethyl-Heptane | | 13475-82-6 | Irritating Odor | 5.20±0.45b | 7.88±0.75a | 2.86±0.27c | - | - | - | - | - | - |
| 38 | 2,4,6-trimethyl-Decane | | 62108-27-4 |  | 16.13±1.39a | 17.14±1.63a | 8.89±0.85b | - | - | - | 1.55±0.18c | - | - |
| 39 | Pentadecane | | 629-62-9 | Waxy | 3.32±0.29a | - | - | - | - | - | 0.40±0.47b | - | - |
| 40 | Hexadecane | | 544-76-3 |  | - | 3.96±0.38b | 4.78±0.46a | - | - | - | - | - | 0.32±0.35c |
| 41 | Heptadecane | | 629-78-7 | Alkane, Biting, Pungent | - | - | 3.95±0.38a | - | - | - | 0.76±0.90c | 0.50±0.43c | 1.60±0.15b |
| 42 | Hexacosane | | 630-01-3 |  | 25.76±2.22b | - | 32.68±3.12a | - | - | - | 11.00±1.34c | - | - |
| 43 | Octadecane | | 593-45-3 |  | - | 1.27±0.12b | 4.13±0.39a | - | - | - | - | - | - |
| 44 | Tritetracontane | | 7098-21-7 |  | - | - | 42.79±4.82a | - | - | - | - | - | - |
| 45 | (-)-trans-Pinane | | 10281-53-5 | Fresh, Pine, Balsam | - | 48.58±4.63a | 17.83±1.71b | - | - | - | - | - | - |
| **Aromatics** | | | | | | | | | | | | | |
| 46 | Dehydro-ar-ionene | | 30364-38-6 | Licorice | 1.90±0.16a | 2.23±0.21a | 0.68±0.65bc | - | - | - | 0.72±0.85b | 0.98±0.83b | 2.06±0.20a |
| 47 | α-Ionene | | 475-03-6 |  | 4.89±0.43a | 1.22±0.12c | 1.65±0.16c | 0.94±0.89c | 1.58±0.15c | 1.94±0.17c | 3.70±0.44b | 1.50±1.28c | 1.51±0.15c |
| 48 | α-Calacorene | | 21391-99-1 | Woody | - | 6.94±0.66a | - | - | - | - | - | - | - |
| 49 | cis-Calamenene | | 72937-55-4 |  | - | 17.04±1.63a | 2.46±0.23b | - | - | - | - | - | - |
| 50 | Cadalin | | 483-78-3 |  | - | 1.13±0.18a | - | - | - | - | - | - | 0.95±0.91b |
| **Heterocycles** | | | | | | | | | | | | | |
| 51 | 2,5-dimethyl-Pyrazine | | 123-32-0 | Chocolate, Nutty | - | 2.52±0.24a | 2.53±0.24a | - | - | - | - | - | - |
| 52 | 2-pentyl-Furan | | 3777-69-3 | Fruity, Green, Floral | 6.00±0.52ab | - | 5.40±0.52b | 1.02±0.97c | - | 6.60±0.63a | - | 0.50±0.43cd | 1.38±0.13c |
| 53 | 2-ethyl-3,5-dimethyl-Pyrazine | | 13925-07-0 | Roasted, Chocolate, Nutty | - | 3.31±0.32b | 8.61±0.83a | - | - | - | - | - | - |
| 54 | 4-Methylthiazole | | 693-95-8 | Green, Nutty, Roasted | 1.68±0.14a | - | 1.22±0.12b | 0.28±0.27d | 1.04±0.96b | 0.75±0.72c | 0.73±0.86c | - | 1.65±0.16a |
| 55 | 1-ethyl-5-methoxy-2-(4-methoxyphenyl)-3-methyl-1H-Indole | |  |  | 12.83±1.14a | - | - | 2.47±0.26b | - | - | 3.24±0.38b | - | - |
| **Alkenes** | | | | | | | | | | | | | |
| 56 | β-Myrcene | | 123-35-3 | Fruity, Sweet, Green | - | 4.11±0.39a | - | - | - | - | - | 0.57±0.48b | - |
| 57 | Limonene | | 5989-27-5 | Citrus, Sweet | 18.98±1.63a | 8.90±0.85b | 1.47±0.15c | - | - | - | - | - | - |
| 58 | trans-β-Ocimene | | 3779-61-1 | Sweet, Herbal | - | 2.56±0.24a | - | - | - | - | - | - | - |
| 59 | β-Ocimene | | 13877-91-3 | Floral, Green, Woody | 11.40±0.99a | - | - | 6.53±0.75b | - | - | - | - | - |
| 60 | 2-Camphene | | 79-92-5 | Woody, Herbal | - | - | - | - | - | - | - | - | 4.54±0.44a |
| 61 | Theaspirane | | 36431-72-8 | Green, Fruity, Honey | 2.16±0.19b | - | - | 0.74±0.85cd | 0.85±0.78c | 4.12±0.38a | 3.86±0.46a | 0.72±0.61cd | 4.57±0.44a |
| 62 | α-Cubebene | | 17699-14-8 | Herbal | - | 4.94±0.47a | 2.67±0.25b | - | - | - | - | - | - |
| 63 | Cubebene | | 13744-15-5 | Citrus, Fruity | - | 2.04±0.19a | 1.86±0.18a | - | - | - | - | - | - |
| 64 | (-)-α-Cedrene | | 469-61-4 | Woody, Cedar, Fresh | - | 6.34±0.64a | - | - | - | - | - | - | - |
| 65 | β-Sesquiphellandrene | | 73744-93-1 | Herbal, Fruity, Woody | - | 4.17±0.40b | 4.86±0.46a | - | - | - | - | - | - |
| 66 | cis-Muurola-3,5-diene | | 157374-44-2 |  | - | 7.14±0.68a | 1.69±0.16b | - | - | - | - | - | - |
| 67 | (E)-β-Farnesene | | 18794-84-8 | Floral | 2.13±0.18a | 2.04±0.19a | - | - | - | - | - | - | - |
| 68 | gamma-Muurolene | | 30021-74-0 | Herbal, Woody, Spice | - | - | 3.40±0.32a | - | - | - | - | - | - |
| 69 | Calarene | | 17334-55-3 |  | - | - | 1.29±0.12a | - | - | - | - | - | - |
| 70 | (+)-epi-Bicyclosesquiphellandrene | | 54324-03-7 | Floral, Sweet, Herbal | - | 6.61±0.64a | 3.10±0.30b | - | - | - | - | - | - |
| 71 | α-Farnesene | | 502-61-4 | Woody, Green, Floral | - | - | - | 8.42±0.98a | 1.14±0.19b | 1.38±0.13b | - | - | - |
| 72 | Caparratriene | | 172549-29-0 |  | - | - | - | - | - | - | - | - | 1.62±0.16a |
| 73 | (+)-δ-Cadinene | | 483-76-1 | Thyme, Herbal, Woody | 1.88±0.16c | 65.17±6.22a | 27.92±2.66b | - | - | - | - | - | - |
| 74 | Cadinadiene | | 29837-12-5 | Spicy, Fruity, Mango | - | 16.32±1.56a | 14.55±1.39b | - | - | - | - | - | - |
| **Alcohols** | | | | | | | | | | | | | |
| 75 | 3-Aminobenzylalcohol | | 1877-77-6 | Roasted, Cocoa Aroma | - | 8.41±0.82b | 14.48±1.38a | - | - | - | - | - | - |
| 76 | cis-Linaloloxide | | 11063-77-7 | Floral | 6.88±0.59a | 3.33±0.32cd | 1.38±0.13e | 1.61±0.19e | 3.91±0.47bc | 4.22±0.39b | 1.04±0.12e | 2.83±0.23d | - |
| 77 | trans-Linalool oxide(furanoid) | | 34995-77-2 | Floral | 5.93±0.52d | 6.38±0.69cd | 2.78±0.27e | 3.49±0.43e | 7.98±0.96bc | 10.91±1.84a | 0.90±0.12f | 8.43±0.70b | 10.39±1.39a |
| 78 | Linalool | | 78-70-6 | Floral, Sweet, Woody, Green | 7.77±0.67cd | 8.75±0.84c | 19.22±1.83a | 4.58±0.48e | 8.27±0.99c | 5.46±0.53de | 16.23±1.83b | 5.28±0.44de | 10.57±1.57c |
| 79 | Maltol | | 118-71-8 | Sweet, Fruity | - | - | - | 42.41±4.90a | 28.29±3.40c | 36.60±3.35b | - | 9.25±0.76e | 17.06±1.76d |
| 80 | (-)-Myrtenol | | 19894-97-4 | Woody, Sweet | - | - | - | 0.78±0.93c | 1.13±0.14c | 4.57±0.42a | - | 2.97±0.25b | 1.15±0.12c |
| 81 | Farnesol | | 4602-84-0 | Fresh, Floral, Sweet | - | - | - | - | 0.99±0.12c | 1.16±0.16bc | 1.33±0.15b | 2.89±0.24a | - |
| 82 | Nerolidol | | 7212-44-4 | Floral, Green, Citrus, Woody | 43.32±3.73a | - | - | 7.24±0.83b | 6.08±0.78b | 6.48±0.64b | - | 2.89±0.24c | - |
| **Esters** | | | | | | | | | | | | | |
| 83 | Methyl salicylate | | 119-36-8 | Sweet, Minty | - | - | - | - | - | - | 2.93±0.33b | 3.31±0.27b | 10.96±1.55a |
| 84 | Butanoic acid, 2-ethyl-, 1,2,3-propanetriyl ester | |  |  | 6.25±0.54c | 1.99±0.19ef | 4.38±0.42d | 2.17±0.25ef | 12.38±1.43a | 8.37±0.76b | 2.94±0.33e | 0.93±0.77f | 2.51±0.25ef |
| 85 | Formic acid, 2-phenylethyl ester | | 104-62-1 | Floral | - | - | - | - | - | - | - | - | 1.75±0.17a |
| 86 | cis-3-Hexenyl hexoate | | 31501-11-8 | Fruity, Green, Waxy | - | 14.27±1.36a | - | - | - | 7.05±0.63b | - | - | - |
| 87 | Methyl 5-acetyl-2-methoxybenzoate | | 39971-36-3 |  | 7.49±0.74b | - | - | 2.19±0.25d | 8.56±0.99b | 13.50±1.21a | 5.42±0.56c | 3.59±0.35d | 8.73±0.84b |
| 88 | Dihydroactinidiolide | | 15356-74-8 | Fruity, Sweet, Woody | 1.55±0.15a | - | - | 0.15±0.15c | 1.17±0.13b | 1.26±0.11b | - | - | - |
| 89 | cis-7-Dodecen-1-yl acetate | | 14959-86-5 | Fruity | - | - | - | - | 1.33±0.15a | - | - | - | - |
| 90 | Phytol acetate | | 10236-16-5 | Waxy, Floral, Fruity, Green | 6.73±0.67b | 21.88±2.88a | 22.82±2.18a | - | - | - | - | - | - |
| 91 | Methyl palmitate | | 112-39-0 | Oily, Waxy, Fatty, Orris | 29.85±2.93a | 5.96±0.57c | 11.31±1.78b | 0.94±0.82d | - | - | - | 1.11±0.15d | 2.22±0.21d |
| 92 | Ethyl palmitate | | 628-97-7 | Waxy, Fruity, Creamy | - | - | 27.91±2.66a | - | - | - | - | - | - |
| **Phenols** | | | | | | | | | | | | | |
| 93 | 2,4-bis(1,1-dimethylethyl)-Phenol | | 96-76-4 | Phenol-Like | - | - | 33.52±3.20a | - | - | - | 6.20±0.64b | - | - |
| **Acids** | | | | | | | | | | | | | |
| 94 | Tetradecanoic acid | | 544-63-8 | Fatty, Waxy | 11.66±1.14b | 3.40±0.32c | 23.92±2.28a | - | - | - | - | - | - |
| 95 | Hexadecanoic acid | | 57-10-3 | Waxy, Fatty | 179.27±17.59b | 271.83±25.94a | 188.30±17.96b | 12.66±1.13c | 7.24±0.84c | - | 21.36±2.19c | 16.82±1.60c | 15.04±1.44c |

Mean values ± SD of three independent experiments were shown.

A: https://www.thegoodscentscompany.com; <https://www.flavornet.org/flavornet.html>.


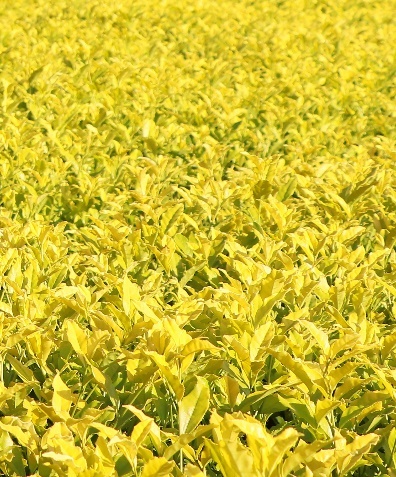

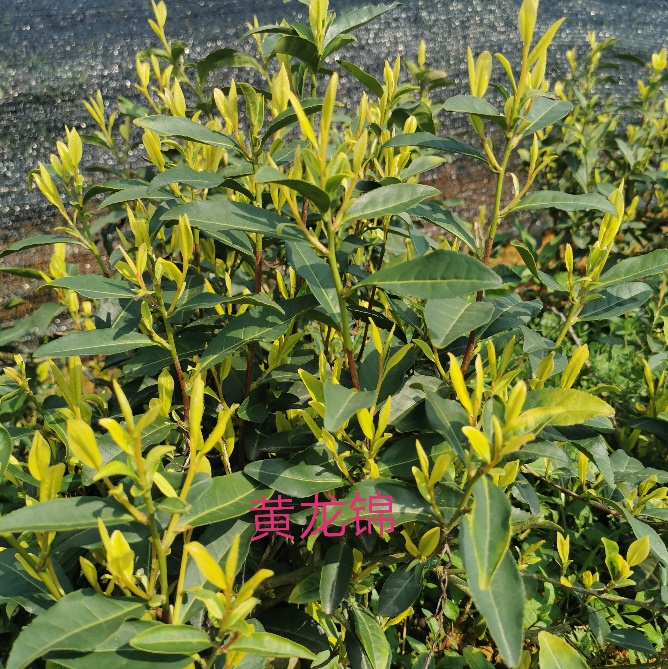

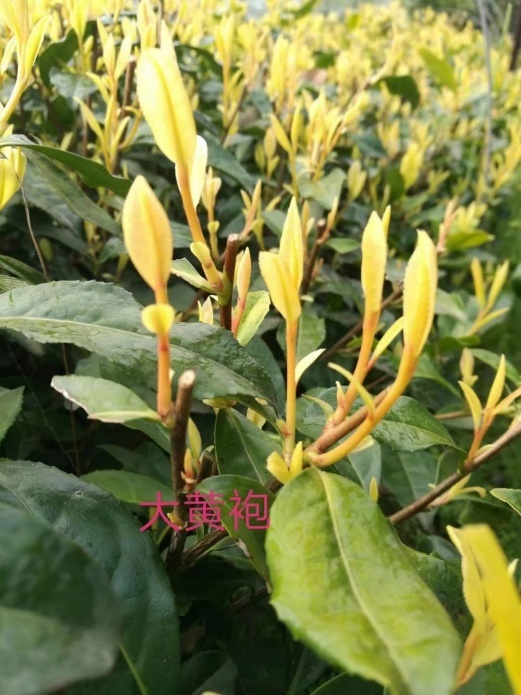


Huangjinya

Huanglongjin

Dahuangpaoya

Fig. S1. The external morphology of Huangjinya, Huanglongjin, and Dahuangpao


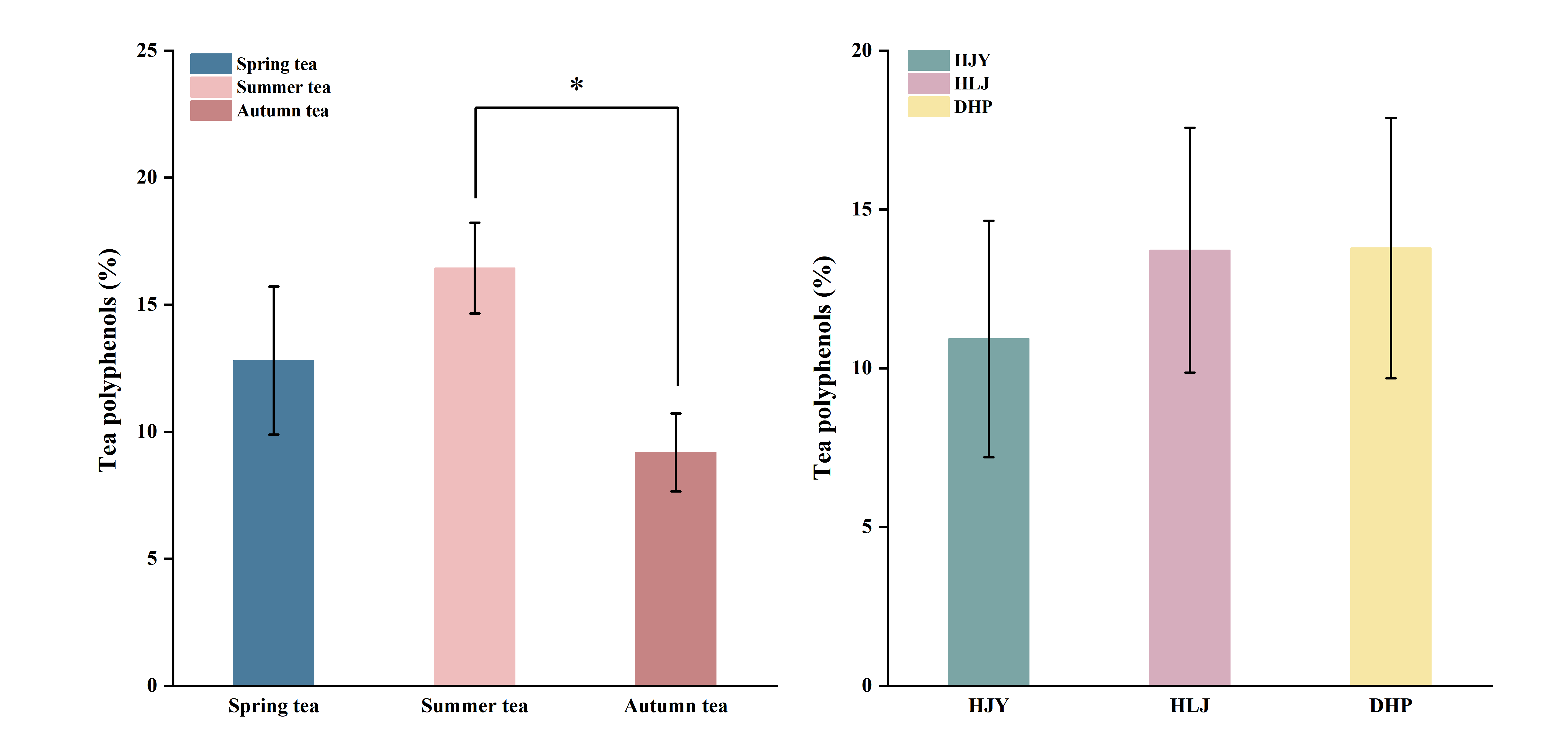


Fig. S2. The content of tea polyphenols in steamed green tea from different etiolated tea cultivars and seasons. * indicates signification differences (*P* < 0.05).
